# Benefiting from binary negations? Verbal negations decrease visual attention and balance its distribution

**DOI:** 10.3389/fpsyg.2024.1451309

**Published:** 2024-11-26

**Authors:** Ngoc Chi Banh, Jan Tünnermann, Katharina J. Rohlfing, Ingrid Scharlau

**Affiliations:** ^1^Faculty of Arts and Humanities, Paderborn University, Paderborn, Germany; ^2^Department of Psychology, Philipps-Universität Marburg, Marburg, Germany

**Keywords:** visual attention, adaptation, linguistic negation, repetition effects, Theory of Visual Attention (TVA), modeling, Bayes

## Abstract

Negated statements require more processing efforts than assertions. However, in certain contexts, repeating negations undergo adaptation, which over time mitigates the effort. Here, we ask whether negations hamper visual processing and whether consecutive repetitions mitigate its influence. We assessed the overall attentional capacity, that is, the available processing resources, and its distribution, the relative weight, quantitatively using the formal Theory of Visual Attention (TVA). We employed a very simple form for negations, binary negations on top of an accuracy-based, TVA-based temporal-order judgment (TOJ) paradigm. Negated instructions, expressing the only alternative to the core supposition, were cognitively demanding, resulting in a loss of attentional capacity in three experiments. The overall attentional capacity recovered gradually but stagnated at a lower level than with assertions, even after many repetitions. Additionally, negations distributed the attention equally between the target and reference stimulus. Repetitions slightly increased the reference stimulus' share of attention. Assertions, on the other hand, shifted the attentional weight toward the target stimulus. Few repetitions slightly decreased the attentional shift toward the target stimulus, many repetitions increased it.

## 1 Introduction

Explanations are not only a scientific issue, but also an everyday phenomenon. The development of machine-learned artificial intelligences (AIs) in recent years, i.e., AIs that are not intrinsically explainable, has increased scientific interest in explanations: What exactly is an explanation? How do good explanations work? How can explanations make the explanatory object more relatable? What mistakes should be avoided when explaining?

Here, we will focus on a crucial, but so far little explored aspect of explanations, the focus of attention. When put into context with objects and actions, misunderstandings can arise when attention is focused on the wrong explanatory object or too little attention is paid to a crucial part of an explanation. In his overview on explanations of AI, Miller (2019) highlights that to mitigate this issue, people do, in fact, frequently stress the relevant causes of an event by giving contrastive explanations (Lipton, 1990; Hitchcock, 1996; Tian and Breheny, 2019). Rather than specifying all necessary and sufficient conditions of why an event occurred, people explain why the event (“fact”) happened in lieu of a counterfactual event (“foil”). The explicitly or implicitly stated counterfactual puts the subject of the explanation into context (Lombrozo, 2012) by implicitly selecting what aspect of the event statement to explain. For instance, “Lisa *closed* the window because it was cold in the bedroom” implies that the closure is being explained, and *not* that Lisa in particular acted or that the window in particular was closed. The fact to be explained can be emphasized by stressing it differently in verbal explanations (or setting it in italics in written explanations). Another method is explicitly negating the non-relevant elements (see above: it is not important that it was Lisa who closed the window) or to state the plausible alternative outcome, that is, the counterfactual (“Lisa *closed* the window, instead of leaving it open, ...”). Generally, counterfactuals are negated events; they are epistemically important for explanations because they restrict the set of states to explain.

Interestingly, cognition research produced evidence that negations hamper early processing (see Tian and Breheny, 2019, and Kaup and Dudschig, 2020, for an overview). Linguistic negations (“Don't touch!”) can lead to higher cognitive demands: They impair encoding, thereby prolonging response times and increasing error rates in sentence and concept verification (e.g. Carpenter and Just, 1975; Wason, 1961; Mayo et al., 2004). Negations can trigger actions toward the opposite (Dudschig and Kaup, 2020b; Gawronski et al., 2008; Wirth et al., 2019), e.g., due to insufficient suppression of the core supposition (ironic process theory, coined by Wegner, 1994) or due to incomplete processing of the whole expression (two-step model, e.g., Clark and Chase, 1972), leading to cognitive conflict. Negations can worsen recall even beyond the negated proposition (Mayo et al., 2014).

From an epistemic point of view, these findings of higher processing costs do not necessarily conflict with the benefits of negations in explanations. Higher processing costs may stem from the function of negations: Negations contrast expectations and point out the unexpected (Glenberg et al., 1999; Kaup et al., 2007; Schneider et al., 2019). Wason (1965) and Glenberg et al. (1999) reported no processing difficulties if negations appear within an appropriate semantic context where the negated question under discussion is plausible.

Expectations may not only emerge from the semantic context but also from statement repetitions. For instance, Rohlfing et al. (2016) posited that in language acquisition, meaning does not emerge from mere verbal label–object mapping but requires this mapping to be embedded in some context that unfolds around a goal or a task. Specifically, a pragmatic frame is established by building expectations through *recurring* social interaction patterns pursuing a joint goal. Such a frame is argued to facilitate a gradual learning process which may be noticeable as performance adaptation. “Opposite day”^1^ as a social practice would get easier to exercise over the course of the day. In explanations this could be a case when multiple alternative hypotheses are refuted (foils)—which corresponds to being exposed to multiple negations.

Adaptation in the sense of lessened processing costs may not be solely attributable to pragmatic understanding but also to more basic cognitive processes. Dudschig and Kaup (2020b) explain adaptation to negations as a result of conflict monitoring—an up-regulation of cognitive control after conflict trials (Botvinick et al., 2001; Deutsch et al., 2006). In their study, processing costs of linguistic negations in a spatial interference task were diminished when the negation was directly preceded by another negation. Similar effects were found in non-linguistic negations as well: Wirth et al. (2019) report that frequent and recent pictorial negations decrease negation effects. Reversal of the negation effects was observed during or after high-frequent and recent negations. To rule out priming effects (which can be present, Mayr et al., 2003), Wirth et al. (2019) combined a two-alternative forced choice task with a classification task which avoided stimulus repetitions. Participants adapted to repeated negations of categories as well.

The conflict monitoring account to adaptation does not compete with the concept of pragmatic frames. In fact, a pragmatic frame is an overarching, nested theory about learning content embedded in a social setting or behavior. It includes a cognitive layer in which conflict monitoring can account for repetition effects as well as a pragmatic layer accounts for pragmatic effects.

Everyday explanatory settings often involve visual stimuli. To assess how effective verbal guidance is in (re-)directing visual attention, it is useful to investigate whether negations—irrespective of their epistemic value—impact visual processing and whether these alterations evolve gradually with repeated exposure.

Past research indicates that a trial involving negations following another alleviates some of the additional processing costs compared to a negated trial following a non-negated trial. Conflict adaptation has been typically analyzed by differentiating whether a trial type (with or without conflict) was repeated (e.g., Schmidt and De Houwer, 2011; Nieuwenhuis et al., 2006; see Dudschig and Kaup, 2020b; Wirth et al., 2019; Dudschig and Kaup, 2018 for negation adaptation). This analysis does not allow us to infer whether the processing costs undergo a *gradual* change beyond a couple of immediate repetitions.

In the following, we assessed whether negations affect visual selection, and if so, whether repetitions mitigate the effects gradually and whether the core supposition (“green” in “not green”) is processed first instead of the entire expression. To do so, a precise tool is required to assess possibly small changes in the amount and distribution of visual attentional capacity between trials. The *Theory of Visual Attention* (TVA) by Bundesen (1990) offers a theoretical account of visual attentional processes and meaningful parameters to quantify visual attention precisely. As we are interested in the perceptual qualities of negations, we used an established TVA-based experimental paradigm; a *temporal-order judgment* (TOJ) task, an unspeeded, accuracy-based visual selection task that is not affected by a motor component. Like the entire TOJ tradition (for an overview, see Spence and Parise, 2010), we prefer judgments because they do not include a speeded motor component. In speeded tasks, the motor component cannot be disambiguated well from the perceptual component due to a possible varying speed-accuracy trade-off between-subject and within-subject. It is possible that—because the judgment is self-paced—it introduces additional noise into the data (for evidence from the auditory domain, see Matthews and Stewart, 2009). However, we accept this because, on the one hand we do not assume that it interferes with the independent variable under investigation, negations. Introducing time windows for the judgment, on the other hand, would contradict both the TOJ tradition and the TVA procedure.

Hereafter, Experiment 1 examines overall attentional capacity under the influence of verbal negations and their repetitions for one to five negations and assertions in a run. Experiment 2 measures both the overall attentional capacity and its distribution across items, hereby allowing to differentiate whether verbal negations hamper visual attention in general or whether the representation of the (non-negated) core supposition has a processing advantage over the (negatively) tagged proposition, showing cognitive control (or, rather a lack hereof). Experiment 3 tests if attentional capacity and attentional weights are able to recover and to match the capacity levels of assertions after up to 220 consecutive repetitions.

### 1.1 General experimental method

The experiments used a TVA-based experimental paradigm (more on that later) with which we estimated the overall attentional capacity and its distribution between the stimuli. Each trial started with a verbal utterance that instructed in an assertive (“now red!”) or negative (“not green!”) manner which of two stimuli were relevant to the subsequent TVA-based selection and judgment task.

Due to the COVID-19 pandemic, all studies were conducted online. Beyond the necessity caused by the pandemic, there is a recent interest to move studies out of the laboratory and “into the wild”—not only due to general participant recruitment considerations but also to gain more external validity in terms of participants and replicability outside of highly artificial settings (Krüger et al., 2021). Loosening the tight control of presentation hardware and the environment raises concerns whether results are still reliable. Reimers and Stewart (2015) ascribe variability in browser experiment presentations mostly to the different PC hardware configurations. Within-system and within-browser presentation variability is small. Software-wise, stimulus presentation duration is relatively precise across most natively running experiment software (i.e., non-browser software such as PsychoPy) and browser-based frameworks, including *jsPsych* that we used in the present study (Bridges et al., 2020).

In practice, various well-known visual effects in speeded response tasks in a *lab, web-in-lab*, and *web* setting were successfully replicated by Semmelmann and Weigelt (2017). Miller et al. (2018) tested three different RT-based cognitive paradigms in a laboratory and “in the wild” over multiple sessions. They concluded that the within- and between-session reliabilities were within a satisfactory range. Another RT-based paradigm conducted on smartphones did not yield lower reliability than on laptops (Pronk et al., 2023). In studies involving our method, TVA-based TOJ tasks, in a *lab* and *web-in-lab* setting with various end devices conducted by Krüger et al. (2021), *web-in-lab* performance correlated with *lab* performance. On average, the estimated overall processing capacity and weights in the *web-in-lab* setting were lower than in a *lab* condition, though. To summarize, conducting browser-based TOJ experiments online is a viable option as presentation is sufficiently precise and effects are sufficiently reliable.

### 1.2 Theory of visual attention

In the following, we outline the relevant aspects of TVA and its linkage to the employed experimental paradigm. TVA views the visual encoding process as a fixed-capacity, independent race (Bundesen and Habekost, 2008, p. 60). The idea of race models, originally proposed by Logan et al. (1984), has become widely used in cognitive psychology (e.g., Logan, 2002) to understand how different cognitive processes compete for resources. The concept of fixed capacity, which is central to attention research, was integrated with race models by Shibuya and Bundesen (1988). TVA's central assumption is that stimuli compete for being encoded into the limited-capacity *visual short-term memory* (VSTM) (usually holding 3–4 objects). Each visual element is assumed to race toward encoding in parallel, without influencing other races. As a result, the processing times are considered mutually independent. The elements share the amount of the common, task-related, and limited processing capacity *C*, the ability to process visual elements within a time period:


(1) $$\begin{array}{l} C=\underset{x\in S}{\sum }v\left(x\right) \end{array}$$

The overall processing capacity is defined as the sum of all individual stimuli's *x* ∈ *S* processing speeds *v*(*x*). Note that only the outcome of the race, not the race itself, can be observed, which manifests as the winning stimulus being encoded or recognized (or, synonymously, *selected*).

According to TVA, the processing speed *v*(*x*) is a result of a two step-process: *(1) Filtering:* Before racing, every stimulus *x* in the visual field is assigned a weight *w*_*x*_, accrued by the sensory evidence η(*x, j*) that *x* belongs to a certain feature category *j* among all categories *R*, and the category's pertinence π_*j*_ to the task.


(2) $$\begin{array}{l} w_{x}=\underset{j\in R}{\sum }\eta \left(x,j\right)\pi_{j} \end{array}$$

Feature categories can comprise, for instance, “pink” or “round”. The sensory evidence of a pink stimulus belonging to the category *pink* would be high, of a red stimulus less so. *(2) Pigeonholing*: Putting the stimulus' individual weight *w*_*x*_ into relation to other stimulus weights, the relative $w_{x}^{*}$ can be derived, which can be considered the “race winning probability” based on sensory properties and task. Consequently, the relative weight $w_{x}^{*}$ of all (task-related) stimuli *x* add up to 1. However, the actual race is not based solely on the stimulus' physical occurrence but also on the response biases β_*i*_ toward one or multiple feature categories *i* ∈ *R*.


(3) $$\begin{array}{l} v\left(x\right)=\underset{i\in R}{\sum }v\left(x,i\right)=\underset{i\in R}{\sum }\eta \left(x,i\right)\cdot \beta_{i}\cdot \overset{\text{Relative weight }w_{x}^{*}}{\overset{︷}{\frac{w_{x}}{\underset{z\in S}{\sum }w_{z}}}} \end{array}$$

The filtering and pigeonholing mechanism draw on early- and late-selection theories (see Bundesen, 1990 and Bundesen and Habekost, 2008, p. 42, for a detailed discussion). The extraction of sensory evidence that “stimulus *x* belongs to category *i*” aligns with late-selection theories, where perceptual categories are not limited to simple physical features like location and color but can involve more complex features, such as alphanumerical identities. In contrast, the general assumption that only selected elements are recognized incorporates principles from early-selection theories. As Bundesen and Habekost (2008) clarify, this approach does not simply combine early- and late-selection theories. Instead, this model offers an integrated perspective, treating “selection and recognition [...] as two aspects of the same process rather than two different stages of processing” (p. 43).

### 1.3 The temporal-order judgment paradigm and linking it to TVA

Visual selection tasks are established methods to estimate TVA's attentional parameters. Typically, TVA parameters are assessed with the letter report paradigms (see Tünnermann et al., 2022 for an overview). We employed a *temporal-order judgment* (TOJ) paradigm, established by Tünnermann et al. (2015) and further developed by Krüger et al. (2016), as the visual selection task. A TOJ trial comprises two stimuli that flicker in brief succession, separated by a *stimulus onset asynchrony* (SOA). The participant then reports in an unspeeded manner the temporal order perceived. Tünnermann et al. (2015) developed the TVA-TOJ model, which explains visual processes in a TOJ task by linking TOJ data with TVA's theoretical considerations (Bundesen, 1990).

In the TOJ paradigm, we relate the processing speed of its two stimuli, a designated probe stimulus p with a reference stimulus r. For all visual stimuli, the probability of a stimulus *x* in a display among other stimuli *S* to be encoded until time *t* is assumed by TVA to follow a exponential saturation curve:


(4) $$\begin{array}{l} F\left(t\right)=\{\begin{array}{cc} 1-e^{-v_{x}\left(t-t_{0}\right)} & \text{if }t>t_{0}\\ 0 & \text{otherwise} \end{array} \end{array}$$

No effective encoding occurs if the stimulus is presented for a shorter time span than the threshold *t*_0_. The probability of encoding depends on the stimulus specific processing speed *v*(*x*), noted in the following as *v*_*x*_. Assuming that a TOJ stems from the stimulus encoding order into the VSTM, Tünnermann et al. (2015) derive the probability of reporting the probe as reaching VSTM first ($P_{p^{\text{1st}}}$) from TVA as follows:


(5) $$\begin{array}{l} P_{p^{\text{1st}}}\left(v_{p},v_{r},\text{SOA}\right)=\{\begin{array}{cc} 1-e^{-v_{\text{p}}\cdot |\text{SOA}|}+e^{-v_{\text{p}}\cdot |\text{SOA}|}\left(\frac{v_{\text{p}}}{v_{\text{p}}+v_{\text{r}}}\right) & \text{SOA}<0\\ e^{-v_{\text{r}}\cdot |\text{SOA}|}\left(\frac{v_{\text{p}}}{v_{\text{p}}+v_{\text{r}}}\right) & \text{SOA}\geq 0 \end{array} \end{array}$$

The SOA denotes probe onset relative to the reference. If the SOA is negative, the probe leads. If the SOA is positive, the probe trails. In case the probe leads, the probe's encoding probability comprises the probability that the probe has been encoded without competing with the reference stimulus, corresponding to Equation 4, and the probability that this has not happened $\left(e^{-v_{\text{p}}\cdot |\text{SOA}|}\right)$ so that the probe competes with the reference and wins with probability $\left(\frac{v_{\text{p}}}{v_{\text{p}}+v_{\text{r}}}\right)$. When the probe trails, it will always compete with the reference, which happens at probability $e^{-v_{\text{r}}\cdot |\text{SOA}|}$ and again, the probe will win the race with probability $\left(\frac{v_{\text{p}}}{v_{\text{p}}+v_{\text{r}}}\right)$. Note that *t*_0_ is not considered in the equation since in our displays it is assumed that all displayed stimuli have the same *t*_0_, and hence, *t*_0_ will cancel out in the equations (see Tünnermann et al., 2015).

For the present work, the overall processing capacity *C* and its relative distribution among the stimuli $w_{\text{p}}^{*}$ are of interest. Therefore, in the following, we re-parameterize the processing speeds in Equation 5 by relating them to the overall processing capacity and the relative weight. Considering the fact that the probe and reference stimulus are the only stimuli in the TOJ task, *v*_*p*_ and *v*_*r*_ sum up to the overall processing capacity *C*.


(6) $$\begin{array}{l} C=v_{\text{p}}+v_{\text{r}} \end{array}$$

In Experiment 1, we employed a target and a distractor task. The involved stimuli were equally relevant to the TOJ. Thus, their processing speed is expected to be equal, and their relative weight can be expected to be shared equally, meaning that $w_{\text{p}}^{*}=w_{\text{r}}^{*}=0.5$, and


(7) $$\begin{array}{l} v_{\text{p}}=v_{\text{r}}=C\cdot 0.5 \end{array}$$

In Experiment 2 and 3, the two stimuli in the TOJ paradigm were similar in every feature except the hue. In these experiments, the probe acts as the point of reference to the task of reporting its order. Nevertheless, both stimuli are technically equally important for the order judgment. From the sensory aspect, η(*x*, red) and η(*y*, green) for *x, y* ∈ *S* can be assumed to be equal, likewise the subjective importance of the feature category β_red (is first)_ and β_green (is first)_. Possible task-related influences on a stimulus' weight (and ultimately on its processing speed) can be accounted for in the pertinence value π in Equation 2. Changes of a category's pertinence are reflected in the relative weights, ultimately dictating the probe's share $w_{\text{p}}^{*}$ in the overall processing capacity *C*.


(8) $$\begin{array}{l} v_{\text{p}}=C\cdot \overset{w_{\text{p}}^{*}}{\overset{︷}{\frac{w_{\text{p}}}{w_{\text{r}}+w_{\text{p}}}}} \end{array}$$

### 1.4 Bayesian parameter estimation

**Figure 1:**
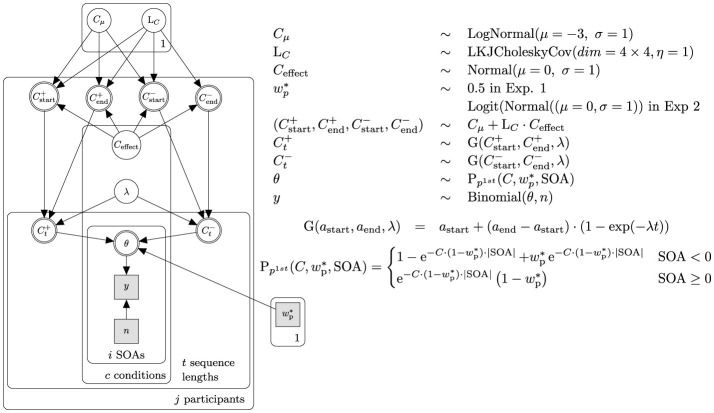
Simplified Bayesian network and priors. The network represents the core structure used in Experiment 1. The likelihood function is represented by θ. The observed ratio of probe-first responses is denoted by *y*, while *n* represents the total number of observations. The processing speed for both stimuli involved in the TOJ is expected to be equal; therefore, the relative attentional weight was held constant at $w_{\text{p}}^{*}=0.5$. For analyses of Experiment 2 and 3, the relative attentional weight $w_{\text{p}}^{*}$ was modeled hierarchically like the overall processing capacity *C*, thereby replacing the constant $w_{\text{p}}^{*}$ node with a copy of the network structure above the θ node. The priors and transformations were adjusted for $w_{\text{p}}^{*}$. Based on the results of an experiment, *C* and $w_{\text{p}}^{*}$ priors for the subsequent experiment were made more informative.

Equipped with the theoretical foundations of TVA-TOJ, we created a hierarchical Bayesian model to estimate parameters. The present model (Figure 1) is adapted from Krüger et al. (2021). We extended the model in the following regards: Firstly, we modeled the data hierarchically to obtain individual and group parameter estimations. Specifically, data is pooled partially (mixed-effects model), which shrinks the variability of estimations (Bayesian shrinkage) (see McElreath, 2020, Chapter 13). Hyper-priors were adapted from Tünnermann (2021) and were updated after each experiment.

Secondly, given the within-subject design which allows estimating correlated parameters, we employed the Lewandowski-Kurowicka-Joe (LKJ) Cholesky covariance prior (Lewandowski et al., 2009) for the varying effects from McElreath (2020, p. 435–443). For Experiment 1, the hyper-parameter η (distinct from TVA's η parameter), was set to 1, leaving the amount of correlation between the attentional parameters in the assertion and negation condition vague. The parameter η was adjusted in the subsequent experiments as we gained more insight about within-subject effect correlations.

Thirdly, we modeled an inverse exponential learning curve to accommodate a gradual adaptation process, acknowledging the bounded nature of attentional parameters. While theoretically, TVA's attentional capacity encompasses positive values, including zero, and could extend to infinity, its practical growth is constrained by the limits of human attention. Likewise, the relative attentional weight, being theoretically bounded at [0, 1], is unlikely to exhibit non-asymptotic growth toward either bound and then abruptly truncate. For Experiment 1, we compared a model featuring a superimposed growth curve to one without, allowing parameter value oscillations at the trial level. To do so, we utilized ArviZ's (Kumar et al., 2019) implementation of an approximated leave-one-out cross-validation with Pareto-smoothed importance sampling (Vehtari et al., 2016). The model incorporating a growth curve provided a better representation of the data, leading us to select it for further evaluation.

Moreover, we added transformations to ensure that sampled values fell within meaningful TVA parameter limits (see Appendix 1).

The models were implemented in pymc (Salvatier et al., 2016) and estimated using the NUTS sampler (Hoffman and Gelman, 2014) with 20,000 samples in four chains, yielding an effective sample size (ESS) of at least 10,000 draws in the parameters of interest (individual attentional capacities, weights, and their means). We report the marginal posterior distribution alongside with the 95% *highest posterior density interval* (HPD) and mode as the point estimate.

## 2 Experiment 1

We aimed to replicate the general consensus that negations incur processing costs and along with the findings reported by Dudschig and Kaup (2020b) that repeating verbal negations leads to adaptation and reduced processing costs. We hypothesize that the effects of negations will be reflected in a lower overall processing capacity and that repeated negations cause the overall processing capacity to gradually recover.

### 2.1 Method

#### 2.1.1 Design

To gain a more fine-grained insight into possibly gradual repetition effects, we incorporated trial sequence lengths as a factor into the experimental design: Participants were presented sequences of 1, 2, and 5 consecutive negation trials or assertion trials. We employed a 2 × 3, within-subject design. Participants were asked to complete at least three sessions with a duration of roughly 10–15 min each on an end device of their choice. Approval from the ethic commission of Paderborn University was obtained.

#### 2.1.2 Participants

Previous experiments that used the TVA-based TOJ paradigm (e.g., Krüger et al., 2021) reached sufficient precision in parameter estimation with 30 participants with a similar number of trials. We therefore aimed for 30 participants as well. The participants were asked to complete at least three identical sessions, each yielding one data set. Incomplete data sets from fewer than three sessions or single session data sets that that could not be matched to a single participant were still considered in analysis as they are still valid and Bayesian analysis respects data according to the varying precision. Thirty volunteers (age: 20–31, *M* = 23.5) were recruited from students and faculty members of Paderborn University and from the recruiting platform *prolific.co*. They participated with or without compensation or received course credits. The breakdown of how many participants completed a certain number of sessions can be found in Appendix 2. The study was performed in accordance with the 1964 Helsinki Declaration. Approval from the ethic commission of Paderborn University was obtained. Participants reported having no color visual deficiencies.

#### 2.1.3 Stimuli

**Figure 2:**
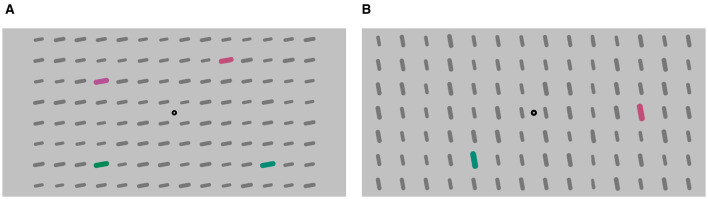
Example of a stimulus displays of **(A)** Experiment 1 and **(B)** Experiment 2 and 3.

Participants were presented with a visual pattern comprising a red and green colored stimuli (Figure 2A). In each trial, a verbal instruction given before the TOJ onset defined the target color and distractor color. The verbal instructions (“now red”, “now green”, “not red”, “not green”) were presented either assertively (“now ...”) or negatively (“not ...”), indicating whether the red or green stimuli were the targets. The audio instructions were delivered by both a synthesized female and male voice. Each instruction consisted of two linguistic items (not & now, red & green) to make the conditions, assertive and negated, comparable in their length and complexity. Both assertive and negated instructions were processed to be of similar length.

**Figure 3:**
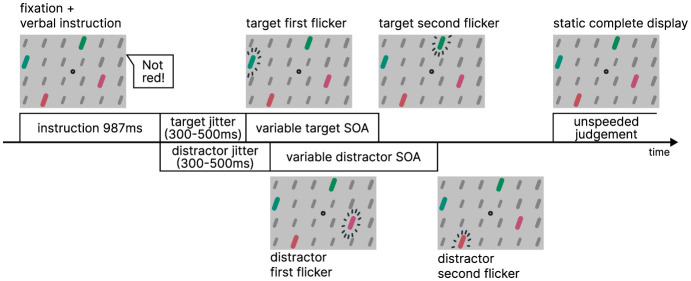
Procedure of Experiment 1.

The display featured a central fixation point against a light gray background (RGB #c1c1c1), surrounded by a 14 × 8 stimulus grid display. The grid consisted of bars which were dark gray background elements (RGB #777777). Within this grid, four bars served as the colored target or distractor pairs. They are arranged in pairs horizontally above each other, resulting in one colored stimulus in each quadrant. The target and distractor pairs were colored either red or green (Figure 2A). Upon revealing the static pattern, a verbal instruction was provided (987 ms). Following the instruction, the pattern remained displayed for an additional duration randomly chosen between 300 and 500 ms. Once the fixation time elapsed, the TOJ task commenced. The target pair flickered with an SOA (strictly speaking, it is a flicker onset asynchrony, not a stimulus onset asynchrony) in seven increments: ±100.0, ±50.0, ±16.7, and 0 ms. The distractor pair flickered with a random SOA after a random fixation time ranging from 300 to 500 ms, independently from the target pair's timings. An example course of a trial is illustrated in Figure 3. Instruction, color, and voice were chosen randomly for each trial, with the color and voice randomized. The negatedness of the expression was controlled as part of the study design.

#### 2.1.4 Apparatus

The study was conducted as an online experiment. The software was written in JavaScript with the open-source framework jsPsych (de Leeuw, 2015), compiled by Babel.js, and bundled by Webpack.js. The behavioral data was managed by JATOS (Lange et al., 2015). Prior to the experiment, the stimuli were loaded into the memory. The participants were free to use any device (ranging from PCs with dedicated periphery to mobile devices such as laptops, tablets, and smartphones), though the use of very large displays, that had a larger visual angle than a 14” laptop screen from normal viewing distance, was discouraged.

#### 2.1.5 Procedure

In each trial, shortly after each grid pattern onset, a voice instructed which color, that is, pair, to attend to (“not green,” “not red,” “now green,” and “now red”). After the display of the TOJ task, participants indicated whether the left or the right bar of the instructed pair flickered first. Judgment was indicated by pressing the Q or the P key, or—in case of using a touch-enabled device—by touching the left or right hand screen half. Participants were instructed to be as accurate as possible. The experiment was self-paced.

Prior to the experiment, participants chose between instructions in English or German language and familiarized themselves with the experiment. The tutorial consisted of 30 trials with larger SOAs in which participants received auditory feedback, indicating the correctness of their responses regarding the direction in which the stimulus of the targeted color first flickered. The main part contained 244 trials consisting of alternating assertion and negation sequences that were 1, 3, or 5 trials long. After roughly 40 trials and always after sequence completion, a break was offered as a break screen. Participants could also take breaks by delaying their answers. One session lasted about 10–15 min.

### 2.2 Results

In the following, we will use the term “first occurrence” for the first trial in a sequence, and refer to subsequent two trials to five trials in sequence as “first repetition” to “fourth repetition”.

**Figure 4:**
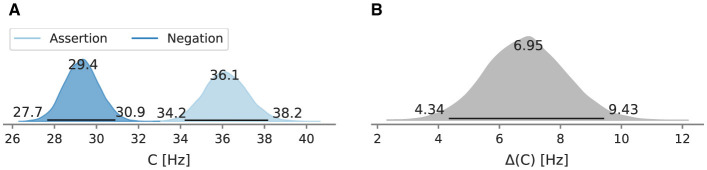
Posterior mean overall processing capacity *C* of Experiment 1 and the difference between conditions. Beside the mode as a point estimate, the HPD is depicted as a black line with its bounds. **(A)** Posterior mean overall processing capacity *C*. **(B)** Difference *C* between the assertion and negation condition.

The full posteriors of the mean overall processing capacity in the main conditions and their difference are depicted in Figures 4A, B. In the assertion condition, the mean processing capacity *C* was estimated at 36.1 Hz [95% HPD: 34.2 Hz, 38.2 Hz]. In the negation condition, the mean processing capacity *C* was estimated at 29.4 Hz [95% HPD: 27.7 Hz, 30.9 Hz]. The HPDs do not overlap. Their difference is 7.0 Hz [95% HPD: 4.3 Hz, 9.4 Hz] and does not include zero.

In traditional psychophysics, the performance of the participants is described by two parameters of the judgment data function (psychometric function). These are discrimination accuracy (difference limen, DL) and the location of the point of subjective simultaneity (“bias”, PSS). The accuracy of the participants was in the normal range for visual TOJs (DL_Assertion_ = 38.36 ms [95% HPD: 36.31 ms, 40.53 ms], DL_Negation_ = 47.20 ms [95% HPD: 44.75 ms, 49.99 ms]). For illustration purposes, the supplementary material presents the aggregated raw data for the experiments and the two conditions each. *C* and $w_{\text{p}}^{*}$ cannot be mapped directly to the traditional measures for TOJs, *difference limen* (DL), and *point of perceived simultaneity* (PSS). We choose the TVA parameters because, as Tünnermann et al. (2015) and Tünnermann (2016), for example, have shown, they are much more meaningful in terms of content (see also Kelber and Ulrich, 2024).

After describing the main conditions and their differences, we now shift focus to the sequential effects observed in the experiment. Specifically, we will analyze how trials are influenced by their position within a sequence, distinguishing between the first occurrence and repetitions.

**Figure 5:**
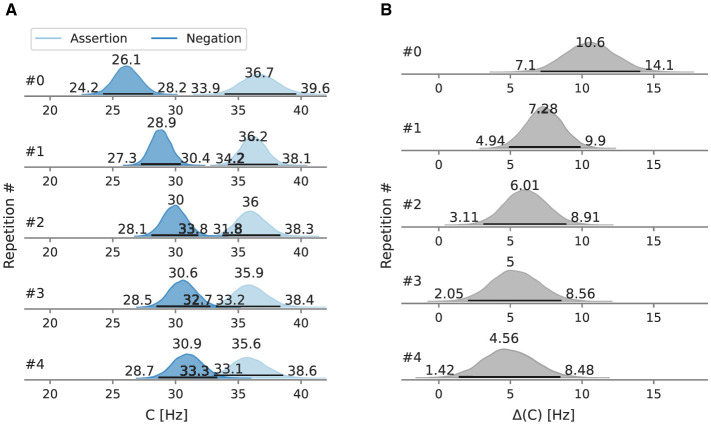
Posterior overall processing capacity *C* of Experiment 1 and the difference between conditions, split by repetitions. Beside the mode as a point estimate, the HPD is depicted as a black line with its bounds. **(A)** Mean *C* split by repetitions. **(B)** Mean *C* difference split by repetitions.

When trials are decomposed into repetitions (Figure 5A), the estimate of the most probable value of the overall processing capacity *C* in the negation condition is always lower than in the assertion condition. At the first negation occurrence, the overall processing capacity *C* is 26.1 Hz [95% HPD: 24.2 Hz, 28.2 Hz], at the fourth repetition at 30.9 Hz [95% HPD: 28.7 Hz, 33.3 Hz]. The *C* estimates increase monotonically with every repetition. The difference between the negation and assertion condition decreases with every repetition. The *C* of the first assertion occurrence is 36.7 Hz [95% HPD: 33.9 Hz, 39.6 Hz]. The difference between posterior distribution of *C* after the forth repetition in the assertion and negation condition does not include zero. The capacity difference in the first occurrence is 10.6 Hz [95% HPD: 7.1 Hz, 14.1 Hz].

Sequences of length 1 and 2 are sub-sequences in sequences of length 5. Sequence of length 1 is a sub-sequence in sequences of length 2. The analyses consider twice as much data in sequence length 2 than in sequence lengths 3–5, and three times as much for sequence length 1.

### 2.3 Discussion

We investigated whether the overall processing capacity is decreased by linguistic negations and whether repeating negations in consecutive trials lessens their negative impact. The results show that linguistic negations strongly reduce the overall visual processing capacity.

The mean difference at 6.8 Hz means that the processing rate is decreased by 6.8 visual elements per second when using negations. At the first occurrence, the decrease of the processing capacity due to negations is larger than 10.6 Hz.

Repetition-wise, the posterior capacity in the first occurrence of a negation is distinctively lower than of the assertion. In the subsequent four repetitions, the capacity increases gradually but does not reach the capacity levels in the assertion condition (Figure 5).

We thus replicated the main findings by Dudschig and Kaup (2020b) that negations incur processing costs, thereby isolating the perceptual component. The unspeeded nature of the TOJ task circumvents possible involvement of motor processes.

So far we have argued that the incurred detrimental effect on processing capacity can attributed to the decreased ability of processing visual stimuli while concurrently processing verbal negations. However, this may not be the sole explanation. Another possible explanation is that the distractor pair could have been erroneously attended to. The distractor pairs' pertinence might have been erroneously set high due to processing the (non-negated) core supposition in the negated instruction first. This would lead to races in which distractors depicting the core supposition are more likely to be encoded into VSTM than the target stimulus pair, thus removing the processing capacity for the actual task. As the distractors have a random SOA, any response to the distractor pair is akin to responding randomly to the actual TOJ task.

In later processing stages where sentence verification and action take place, these two possible phenomena, a central processing bottleneck (Pashler, 1994) and an incomplete processing of the supposition, are well-known: Linguistic negations are hypothesized to be subject to either a one-step process or a two-step process (see Mayo et al., 2004, for an overview). In the two-step model, negated propositions (“not green!”) are decomposed into a non-negated core supposition and a negation tag. The two parts are processed sequentially (Clark and Chase, 1972) instead of being perceived as one meaningful assertive unit (one-step model; e.g., MacDonald and Just, 1989). The two-step model attributes possible negation effects toward the proposition's opposite to a mental representation of the core supposition and the subsequent processing (e.g., Mayo et al., 2004) or act on it (e.g., Kaup and Dudschig, 2020) without considering the negation tag.

The design of Experiment 1 did not allow differentiating the two possible phenomena. In the following experiment, we modified the design so that the overall processing capacity and “erroneous” shifts of attention can be accounted for.

## 3 Experiment 2

Experiment 1 demonstrated that verbal negation reduces visual processing capacity and showed incomplete adaptation to negations after several repetitions. It is unclear whether the reduction of the overall processing capacity in the previous experiment resulted from processing the verbal negation or from attending the wrong stimuli. To distinguish both phenomena, we transformed the TOJ task into one that requires attending to and reporting a particular colored stimulus. This design allows estimating the attentional weight beside the processing capacity.

We expect one of three possible outcomes for the negation condition: The overall processing capacity *C* is decreased but the attentional weight $w_{\text{p}}^{*}$ does not change between conditions, only the attentional weight $w_{\text{p}}^{*}$ is affected but not the processing capacity *C*, or both attentional parameters are affected. A decrease in *C* indicates concurrent negation processing that locks up cognitive resources. Changes in the relative attentional weight $w_{\text{p}}^{*}$ reflect the distribution of attention. A lower $w_{\text{p}}^{*}$ in the negation condition means that the distractor color received more processing resources than in the assertion condition. In absolute terms, a relative attentional weight below 0.5 indicates that the stimulus representing the core supposition is favored over the probe in early processing.

### 3.1 Method

#### 3.1.1 Participants

We aimed for 42 complete data sets, each consisting of three sessions. The estimation of an additional parameter required more participants to reach higher posterior precision. The sample size was predefined. In total, 42 volunteers with a complete data set were recruited from prolific.co (19–50, M = 29.1), a further three dropped out before completing the data set. The breakdown of how many participants completed a certain number of sessions can be found in Appendix 2. One participant who completed four sessions had to retake the first session due to reported misunderstanding of the task, resulting in the exclusion of their initial session from further analyses. In Appendix 2, this participant is listed as having completed four sessions. A further participant with one session who reported misunderstanding the task and a further participant who reported color vision deficiencies was excluded from further analyses.

The present experiment is a re-conduction of an earlier study in which concerns about the intelligibility of the female English voice were raised. The data of this earlier study can be found in the supplementary material. They are partially at chance level and thus, substantially different from the analysis of the present Experiment 2.

#### 3.1.2 Stimuli

Stimuli were the same as in Experiment 1, except that there was only one pair of stimuli (Figure 2B). One stimulus is red and the other green.

#### 3.1.3 Procedure and apparatus

After the auditory instruction and TOJ task onset, participants judged whether the verbally instructed color flickered first by pressing the Q key or whether it flickered second by pressing the P key. Touch device users responded by tapping on the left or right screen half, respectively. The mapping was counterbalanced.

The apparatus used is identical to that in Experiment 1.

### 3.2 Results

We report first the overall difference between negations and assertions and will look into repetition effects thereafter.

**Figure 6:**
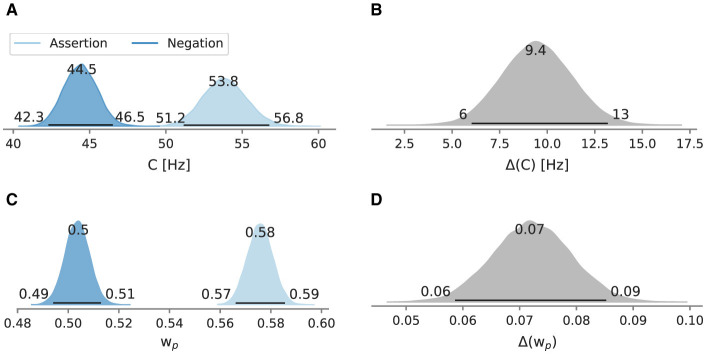
Posterior mean overall processing capacity *C* and attentional weight $w_{\text{p}}^{*}$ across repetitions of Experiment 2 and their differences. Besides the mode as a point estimate, the HPD is depicted as a black line with its bounds. **(A)** Mean *C* across repetitions and **(B)** the difference between conditions. **(C)** Mean $w_{\text{p}}^{*}$ across repetitions and **(D)** the difference between conditions.

Figure 6 depicts the posterior mean attentional capacity and relative weight across participants and repetitions and their difference. In the assertion condition, the mean overall processing capacity was at 53.8 Hz [95% HPD: 51.2 Hz, 56.8 Hz]. In the negation condition, the processing capacity was decreased (44.5 Hz [95% HPD: 42.3 Hz, 46.5 Hz]). In the assertion condition, the mean relative attentional weight on the probe $w_{\text{p}}^{*}$ was estimated at 0.576 [95% HPD: 0.566, 0.586]. The mean relative attentional weight $w_{\text{p}}^{*}$ in the negation condition was estimated at 0.504 [95% HPD: 0.494, 0.513]. A relative weight of $w_{\text{p}}^{*}=0.5$ indicates that attention is equally distributed among probe and reference. A $w_{\text{p}}^{*}$ larger than 0.5 indicates that the probe received a larger share of the overall processing capacity whereas a $w_{\text{p}}^{*}$ smaller than 0.5 indicates the opposite. The HPDs of the posterior distributions for both attentional parameters do not overlap. All differences are larger than zero. In the assertion condition, the mean difference limen was 26.7 ms [95% HPD: 25.4 ms, 28.2 ms], whereas in the negation condition, the mean discrimination ability was less accurate at 31.2 ms [95% HPD: 29.8 ms, 32.8 ms]. The point of perceived simultaneity was 6.1 ms [95% HPD: 5.3 ms, 7.1 ms] in the assertion condition, while in the negation condition, it was 0.4 ms [95% HPD: −0.6 ms, 1.2 ms], overlapping 0 ms.

**Figure 7:**
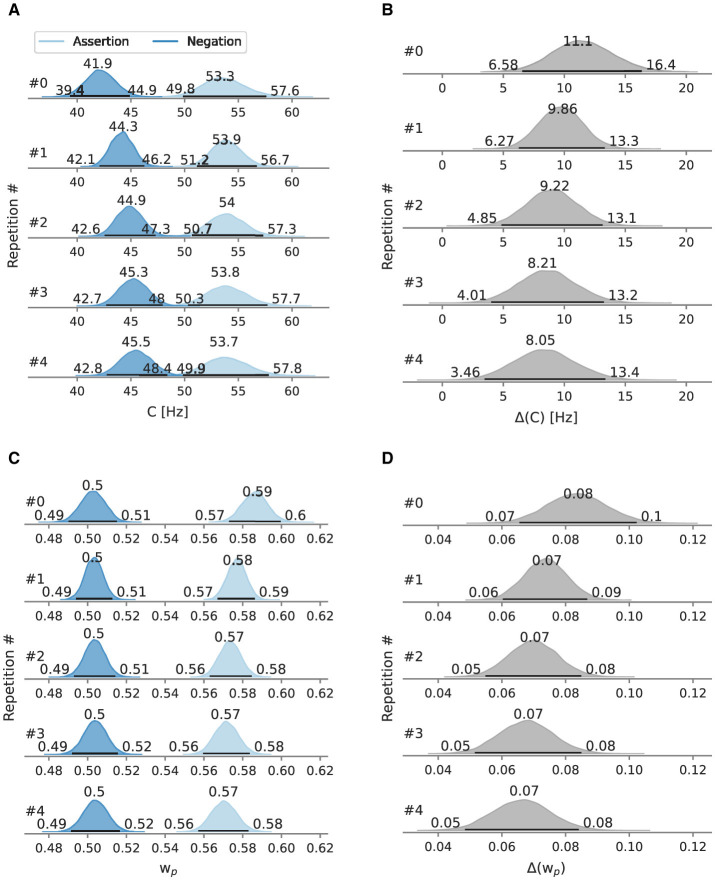
Posterior overall processing capacity *C* and attentional weight $w_{\text{p}}^{*}$ of Experiment 2, split by repetitions. Besides the mode as a point estimate, the HPD is depicted as a black line with its bounds. **(A)** Depicts *C* split by repetitions and **(B)** the difference between conditions. **(C)** Depicts $w_{\text{p}}^{*}$ split by repetitions and **(D)** the difference between conditions.

Figure 7 shows the attentional parameters broken down into repetitions and their difference rank-wise. Similar to Experiment 1, as sequences of length 1 and 2 are sub-sequences in sequences of length 5, respectively 2, the analyses consider twice as much data in sequence length 2 than in sequence lengths 3–5, and three times as much for sequence length 1.

At the first negation, the processing capacity *C* was estimated at 41.9 Hz [95% HPD: 39.4 Hz, 44.9 Hz]. At the first assertion, *C* is at 53.3 Hz [95% HPD: 49.8 Hz, 57.6 Hz]. The marginal likelihood difference between the two conditions peaks at 11.1 Hz. In the negation condition, the processing capacity increases with the number of repetitions, reaching 45.5 Hz [95% HPD: 42.8 Hz, 48.4 Hz] at the fifth negation. In the assertion condition, starting from the second assertion, *C* stagnates at 53.9 Hz with slightly varying HPD interval limits and modes. The difference between both conditions remains larger than zero even at the fifth repetition. However, the difference between both conditions decreases with every repetition, driven by *C* in the negation condition.

In the first occurrence in the negation condition, the probe has an attentional weight $w_{\text{p}}^{*}$ of 0.504 [95% HPD: 0.490, 0.515]. The weight does almost not change throughout the repetitions (0.503 at the last repetition). In the assertion condition, $w_{\text{p}}^{*}$ starts with 0.588 [95% HPD: 0.573, 0.599] and decreases over time, reaching 0.570 [95% HPD: 0.557, 0.583] at the fourth repetition.

The $w_{\text{p}}^{*}$ differences between both conditions also have a negative trend, which is driven by the weights of the assertion condition.

### 3.3 Discussion

Compared to the assertion condition, the overall processing capacity *C* in the negated condition was reduced (distinctively more than in Experiment 1), implying that some cognitive resources that affect visual processing capacity were taken for negation processing. Furthermore, assertions shifted the attentional weight by 0.071 toward the probe. The consequence is that in the assertion condition, the probe gains a larger share of the attention than the reference. Negations caused the processing capacity to be shared equally between target stimulus and reference stimulus. The equal distribution of the processing capacity indicates that a share of attention went toward the core supposition, represented by the reference stimulus. Given that understanding the target color in the assertion condition can strongly bias toward the probe, one possible explanation of the balanced resource distribution could be occasional misdirection toward the distractor color. However, for this conclusion to be confidently drawn, it would require the reference stimulus to be favored over the target stimulus, resulting in $w_{\text{p}}^{*}<0.5$. Another possibility is that negation processing as one single unit was more often than not incomplete. Participants may have made a TOJ without being aware of the final proposition and put together the auditory instructions with their TOJ after the flickering. Such a strategy change may be more feasible in this experimental setting compared to the previous experiment. The former comprised an additional pair of flickering distractors, that is, four stimuli instead of two as in the latter experiment.

Sequential effects of negated instructions on the overall processing capacity were present and on the attentional weight absent. Still, after five consecutive repetitions, negations seem to have a detrimental effect on the overall processing capacity. We do not know if more repetitions are needed to meet parameter levels as in the assertion condition.

## 4 Experiment 3

Within five repetitions in Experiment 1 and 2 we observed some adaptation to negations and no full recovery of attentional parameters. If a full adaptation to negations is possible, it remains elusive due to its slow progression and relatively few repetitions. In the following experiment, using a sliding window approach, we analyzed a very large number of repetitions in a blocked design to estimate whether the attentional parameters in the negation condition gradually meet the levels as in the assertive condition. To our knowledge, no studies have explored the effects of long negation sequences on attention. Consequently, we cannot deduce how many repetitions may be necessary for full recovery. We consider it is reasonable to assume that 220 consecutive repetitions should suffice to get an impression whether there is a full adaptation in a practically relevant time frame.

### 4.1 Method

#### 4.1.1 Design

We employed a one-factor, within-subject design. Negation and assertion trials were blocked and completed in separate sessions. The order in which participants completed the blocks was randomized.

#### 4.1.2 Participants

We recruited 30 volunteers (18–52, M = 25.7) with a full data set (that is, each one block with negations and assertions) from Paderborn University and prolific.co. A further five participants were excluded because they provided data for only one condition and a further participant who reported color vision deficiencies was excluded. Similar to Experiment 2, further 15 participants were excluded mid-data collection irrespective of the results due to concerns regarding the auditory stimuli's intelligibility of the female English voice. We could not prevent this because of the overlapping run with Experiment 2. The data can be found in the supplementary material.

#### 4.1.3 Stimuli and procedure

The stimuli and procedure were the same as in Experiment 2. Stimuli flickered with additional two increments of SOAs (±66.7 ms, ±33.37 ms) to increase precision of the estimation for possibly small differences, yielding 220 trials in each block. Participants completed one block each with negated and assertive instructions. The order of the blocks and the key mapping was counterbalanced. Participants were not informed that each block consisted solely of assertions or negations.

### 4.2 Results

**Figure 8:**
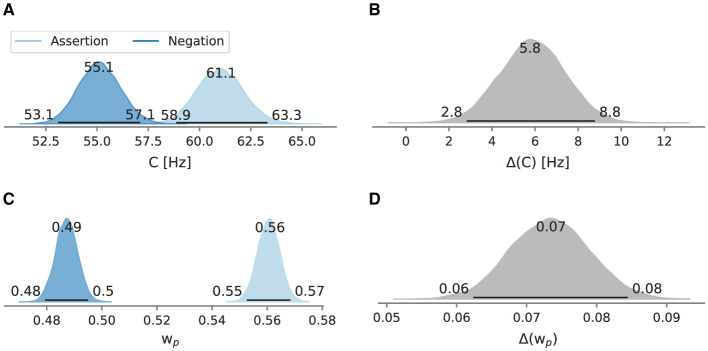
Posterior mean attentional parameters in Experiment 3. Besides the mode as a point estimate, the HPD is depicted as a black line with its bounds. **(A)** Mean *C* across windows. **(B)** Mean *C* difference between assertion and negation condition. **(C)** Mean $w_{\text{p}}^{*}$ across windows. **(D)** Mean $w_{\text{p}}^{*}$ difference between assertion and negation condition.

In the negation condition, the mean processing capacity *C* at 55.1 Hz [95% HPD: 53.1 Hz, 57.1 Hz] is lower than in the assertion condition, where *C* is at 61.1 Hz [95% HPD: 58.9 Hz, 63.3 Hz] (Figure 8A). Likewise, relative attentional weight $w_{\text{p}}^{*}$ in the negation condition (0.487 [95% HPD: 0.479, 0.495]) is lower than in the assertion condition (0.561 [95% HPD: 0.553, 0.568], Figure 8B). Neither difference between the main conditions includes zero (5.8 Hz [95% HPD: 2.8 Hz, 8.8 Hz]; 0.073 [95% HPD: 0.062, 0.084]) (Figures 8C, D).

In the assertion condition, the mean difference limen was 23.3 ms [95% HPD: 22.5 ms, 24.2 ms], whereas in the negation condition, the mean discrimination ability was less accurate at 25.2 ms [95% HPD: 24.3 ms, 26.1 ms]. The point of perceived simultaneity was 4.3 ms [95% HPD: 3.7 ms, 4.9 ms] in the assertion condition, while in the negation condition, it was negative at −0.9 ms [95% HPD: −1.5 ms, −0.4 ms].

**Figure 9:**
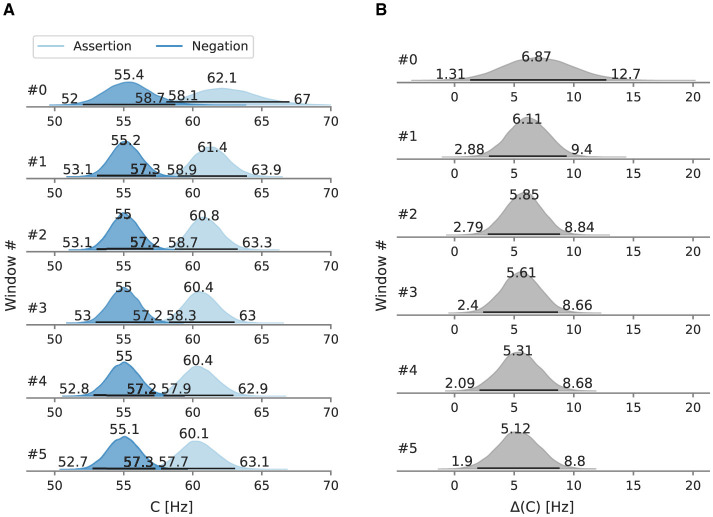
Posterior processing capacity *C* in Experiment 3, evaluated in sliding windows of 110 trials. Besides the mode as a point estimate, the HPD is depicted as a black line with its bounds. **(A)** Overall processing capacity *C* split into windows. **(B)** Difference of the overall processing capacity *C* split into windows.

**Figure 10:**
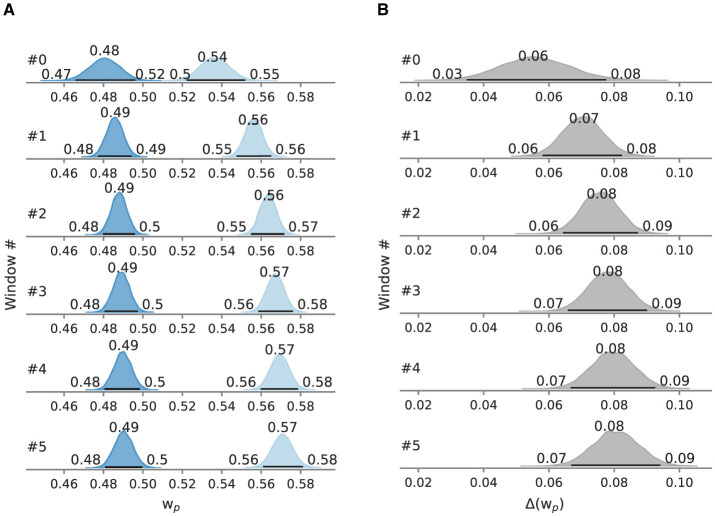
Posterior attentional weight $w_{\text{p}}^{*}$ in Experiment 3, evaluated in sliding windows of 110 trials. Besides the mode as a point estimate, the HPD is depicted as a black line with its bounds. **(A)** Relative attentional weights $w_{\text{p}}^{*}$ split into windows. **(B)** Difference of the relative attentional weights $w_{\text{p}}^{*}$ split into windows.

To test for gradual changes in capacity and weight, we analyzed the data in a sliding window of 110 trials in size and with 22 trials between the next window, resulting in six data windows. When decomposed into sliding windows (Figures 9, 10), in the negation condition *C* remained in the 55.1–55.4 Hz range and $w_{\text{p}}^{*}$ in the 0.480–0.490 range. The processing capacity under assertions decreases over the course of the experiment; starting at 62.1 Hz [95% HPD: 58.1 Hz, 67.0 Hz] and ending at 60.1 Hz [95% HPD: 57.7 Hz, 63.1 Hz]. The relative attentional weight $w_{\text{p}}^{*}$ increased from 0.535 [95% HPD: 0.522, 0.552] to 0.571 [95% HPD: 0.561, 0.581]. The *C* difference decreases and the $w_{\text{p}}^{*}$ difference increases as the experiment progressed, mainly driven by parameter changes in the assertion condition.

### 4.3 Discussion

We replicated the main effect of negations from Experiment 1 and 2—negations were detrimental for the overall attentional capacity. Like in Experiment 2, while assertions shifted the attentional weight $w_{\text{p}}^{*}$ considerably toward the probe, negations distributed attention more equally between probe and reference. Here, reference is favored slightly.

After decomposing a block into multiple sliding windows of 110 trials, the posteriors show that repeating negations seem to fail to recover the processing capacity *C* and the attentional weight $w_{\text{p}}^{*}$ to levels in the assertion condition. The *C* difference gets smaller over time due to assertions decreasing *C* gradually and not negations increasing *C*. The attentional weight difference increases over time due to an increasing weight shift toward the probe in the assertion condition.

To conclude, negation effects persisted which is in line with Dudschig and Kaup (2020a). Dudschig and Kaup (2020a) showed that even after a long preparatory period, a negation effect still remained. In their experimental paradigm, the valence of the upcoming trial was shown to participants. Participants responded to subsequent directional instructions. Preparatory time up to 1, 500 ms was not sufficient for full adaption in both response time and error rate in linguistic negation.

In our study, there was little adaptation to negations. The small changes observed between the assertion and negation conditions were mainly driven by variations in the assertion condition. Notably, even with (1) a non-speeded judgment task and (2) blocks with 220 trials of the same type, participant did not adapt to negations fully. The results indicate an inescapable impact of negations on visual attention, leading to reduced processing capacity and a more balanced distribution of attention.

## 5 General discussion

We examined the influence of repeated verbal negated instruction on TVA's overall processing capacity and its distribution. We compared these with the attentional parameters under assertions in three experiments.

We addressed the question whether negations reduce the available overall processing capacity and whether negations change the distribution of attention $w_{\text{p}}^{*}$ between the instructed target and the reference. With this, we introduced a novel dependent variable into the research on verbal guidance that is valid for settings involving objects and actions. We hypothesized that consecutive repetitions of negated propositions gradually recover processing capacity *C* and gradually shifts the relative attentional weight $w_{\text{p}}^{*}$ toward the target.

Three experiments consistently show that negations are detrimental to the overall processing capacity *C*. The processing capacity increased gradually after multiple repetitions but nowhere near to capacity with assertions. With a mean reduction of 6 Hz based on capacities of around 61 Hz, the reduction is quite substantial. Instead of processing 61 elements of this kind per second, participants would only be able to proceed 55 items per second after negations. After repeating negations 220 times consecutively, it seems to have reached a plateau that is well below the processing capacity with repeated assertions—more precisely 55 Hz. In Experiments 1 and 2, where alternations between negations and assertions occurred very often, the first negation after an assertion penalizes the overall processing capacity by ≈10 Hz. This effect is in the same magnitude as riding a bicycle in a simulated high- versus low-traffic environment (Stratmann et al., 2019).

Shifting focus from the impact on overall processing capacity, we now discuss how negations influence the distribution of attentional resources. Negations distributed the attentional resources almost evenly between target and reference, maintaining a constant weight across up to four repetitions. Upon analyzing 110 consecutive negations, we observed a slight preference for the reference over the probe. Notably, even after 220 consecutive negations, this slight preference persisted. Assertions directed a larger share $w_{\text{p}}^{*}$ of the higher overall processing capacity to the probe. When assertions and negations were alternated very often, the attention was diverted to the probe by 0.07. This level of attentional weight change is akin to the effect observed comparing a colored stimulus surrounded by elements of its complementary color to a stimulus surrounded by elements of the same color, suggesting a comparable impact on where attention is directed (Krüger et al., 2021). When assertions were repeated up to five times, there was a gradual shift toward a more balanced distribution of attentional weight. However, in the long run, the opposite trend emerged: the target gained more attentional weight.

Overall, both the mean overall processing capacity and the attentional weight vary considerably across experiments. This is also known from other studies (Habekost, 2015; Krüger et al., 2021). This variability becomes particularly apparent when using TVA which provides substantive, quantitatively interpretable parameters. In contrast, many other studies report only statistical effects on reaction times or accuracy data (see e.g., Krüger et al., 2018; Tünnermann and Scharlau, 2021 for a discussion). A simple post-hoc comparison of processing capacities and attentional weights across experiments yielded a mean capacity difference of 17.7 Hz (assertion) and 15.1 Hz (negation) between Experiment 1 and 2, and a further increase of 7.3 Hz (assertion) and 10.6 Hz (negation) between Experiment 2 and 3. The differences between 1 and 2 may be explained by a present distractor pair. On the other hand, 2 and 3 were visually identical. Furthermore, between Experiment 2 and 3, the trends in the parameters reversed. In Experiment 2, the overall processing capacity *C* in the negation condition increased, approaching the constant levels of *C* observed in the assertion condition. In Experiment 3, the trend was changed: The overall processing capacity in the negation condition remained relatively constant, while *C* in the assertion condition approached the former. The trend of the attentional weight $w_{\text{p}}^{*}$ specifically in the assertion condition also reversed. In Experiment 2, $w_{\text{p}}^{*}$ in assertions approached 0.5. However, in Experiment 3, $w_{\text{p}}^{*}$ diverged from 0.5. This variability clearly requires further investigation. To date, little research has addressed this issue (with some exceptions, see Künstler et al., 2018; Poth et al., 2014; Biermeier et al., 2024). We cannot resolve this question within the scope of this paper.

It is conceivable that the two parameters, *C* and $w_{\text{p}}^{*}$, may correlate and thus not be entirely independent. This correlation could make it challenging to distinguish between the two mechanisms by which negations might affect visual processing: either generally blocking visual processing through a central bottleneck, as indicated by a reduction in *C*, or merely directing attention, as reflected by a shift in $w_{\text{p}}^{*}$. TVA fundamentally assumes that *C* and $w_{\text{p}}^{*}$ are not correlated. Finke et al. (2005) examined whether overall processing capacity and the difference in relative weights for distractors compared to targets α, along with other TVA parameters, are empirically independent. Using a partial report task, they found a very low and non-significant correlation (Pearson product-moment correlation coefficient *r* = −0.04) between the two parameters. In Experiment 2, the correlation between the posterior overall processing capacity and relative attentional weight was mostly very weak (|*r*| < 0.1, see Appendix 3) and weak at most (0.1 ≤ *r* < 0.34). Potential inter-parameter correlations could be an artifact of Monte-Carlo Markov chain-based parameter estimation. We mitigated auto-correlations, that may mediate inter-parameter correlations, by extending the sampling process and reaching an effective sample size of at least 10,000 samples. Nevertheless, without systematic investigations, we cannot exclude the possibility that correlations between parameters are not merely phenomenological but might result from a modeling artifact. For TOJ, there are no general studies on correlations that we are aware of. However, Krüger et al. (2016) successfully varied $w_{\text{p}}^{*}$ systematically using salience manipulations without affecting *C*, while employing the TVA-TOJ model as in the present paper. If *C* and $w_{\text{p}}^{*}$ do empirically correlate, the possible explanations for negation effects on attention—where a reduction in overall processing capacity indicates a lock-up of processing resources and a shift in the attentional weight indicates a redistribution of these resources—remain valid given interpretability of TVA parameters.

Assuming that the $w_{\text{p}}^{*}$ changes are of phenomenological nature, it remains unclear whether the balanced distribution of attention results from occasional misdirection of attention to the non-negated supposition or also from an overall strategy change (see discussion in Experiment 2). The mechanism may be dependent on the specific task and visual set-up. To better understand these mechanisms, future studies could implement experimental manipulations that independently target the effects on these two mechanisms. For example, negating the underlying TOJ instruction (e.g., “now first” or “not second”) could help determine if there is a broader strategic shift in attention.

In this study, we pushed the boundaries of TVA: This investigation diverges from typical TVA studies, as it delves into overall processing capacity variability, an aspect not traditionally addressed within TVA framework. Prior research, such as that by Poth et al. (2014) and Künstler et al. (2018), has demonstrated variability in capacity, revealing phenomena not thoroughly explained by TVA. These studies observed a reduction of *C* when participants worked on a secondary task that either involved continuous motor activity or visual monitoring. Unlike previous works, our study focused on processing different expressions of an instruction. The changes in *C* observed in our study cannot be explained by the change of stimuli as the visual task remained constant. It seems that processing of certain instructions, therefore the working memory load, influenced the available overall processing capacity.

Outside of TVA, the notion that pragmatics and working memory load influences the cognition and performance in domains including the visual domain is well-established (e.g., in negation research). A comparable finding to a somewhat similar instruction set to ours is a study by Orenes et al. (2014). They examined overt attention toward verbally announced stimuli in a binary negation presentation. Even without the necessity to pay attention to the announced stimulus to solve the subsequent task, the probability of fixating both the probe stimulus and the alternative was closer together in the negation condition than in the assertion condition (where the probability of fixating the alternative was rather close to zero). There seems to be a sustained interest in this domain. It may be worthwhile to formalize the influence of external factors on TVA and to link it with collected data. Future research could explore executive control of TVA (Logan and Gordon, 2001), an extension of TVA which accounts for concurrent, executive processes in dual-task situations that can manipulate TVA parameters. While our paradigm does not strictly involve dual-tasking, Logan and Gordon's (2001) theory extension offers a starting point for modeling and understanding findings such as ours and those of Künstler et al. (2018) and Poth et al. (2014). Despite its potential, executive control of TVA has yet to gain significant traction in the field.

Returning to pragmatic frames, the general context of negating everything (which can be feasibly interpreted as a social practice akin Opposite Day) may already qualify as a pragmatic context. However, simply repeating negations do not fully alleviate the negative effects they induce. It is conceivable that a pragmatic context for framing specifically necessitates a constant target, (to some degree) irrespective of the exact expression to facilitate learning.

In summary, the effect of negated instructions on processing costs could be replicated: Negations had a pronounced detrimental impact on TVA's overall visual processing capacity *C*. Repeating negations across multiple trials resulted in a negligible increase in *C*, insufficient to reach the levels observed in the assertion condition, even after 220 consecutive trials. Negations distributed attention almost equally between target and reference, with a slight tendency toward the reference, whereas assertions shifted the processing resources considerably toward the target. In contexts lacking a meaningful background or a “larger picture”, binary negations should used rather sparingly to point out a certain visual target. Conversely, negations can be effective in explanatory settings where attention needs to be drawn to both options.

## Data Availability

The data and analysis code for all experiments are available at https://github.com/PsyLab-UPB/jspsych-toj-experiments (experiments) https://osf.io/wuh4s/?view_only=92fba4c0efa042c89ec55c421b8431aa (data and analyses). None of the experiments were preregistered.

## Footnotes

^1^ https://en.wikipedia.org/wiki/Opposite_Day
