# Supplementary material for: Benefiting from binary negations? Verbal negations decrease visual attention and balance its distribution
Source: Front Psychol. 2024 Nov 26;15:1451309. doi: 10.3389/fpsyg.2024.1451309 (PMC11628264; doi:10.3389/fpsyg.2024.1451309)
Supplement: Supplementary file 1 [file Data_Sheet_1.pdf]

Supplementary Material

1 BAYESIAN PARAMETER ESTIMATION

In order to prevent sampling from invalid values within the normally distributed hyper-priors, normal distributions for C were log-transformed to ensure positive values. Additionally, the relative attentional weight w_p^* was subjected to a logit transformation to confine its values within the interval $[0, 1]$.

As the attentional parameters are modeled as an conditional effect added to a common population mean, including more data in the analysis makes the posterior more precise without biasing them due to the data's imbalance. The *highest posterior density interval* (HPD) intervals for shorter sequence lengths (where more data points are available) thus can be smaller in the model without superimposed adaptation curves. This does not apply to the model with the superimposed adaptation curve; all data points contribute to the common estimation of the growth curve parameters.

2 NUMBER OF COMPLETED SESSIONS AND PARTICIPANT COUNT IN EXPERIMENT 1 & 2

**Completed sessions**	**Participant count**	
	**Exp. 1**	**Exp. 2**
5	3	–
4	1	1
3	27	41
2	1	–
1	2	2

3 INTERPARAMETER CORRELATIONS (C AND w_p^*) IN EXPERIMENT 2 & 3

**Experiment**	**Condition**	**Repetition**	**Pearson's r**
Exp. 2	Assertion	0	0.185
		1	0.117
		2	0.100
		3	0.095
		4	0.089
	Negation	0	-0.074
		1	-0.035
		2	-0.017
		3	-0.008
		4	-0.005
	Assertion & Negation	0–4	0.191

**Experiment**	**Condition**	**Window**	**Pearson's r**
Exp. 3	Assertion	0	0.216
		1	0.328
		2	0.336
		3	0.331
		4	0.324
		5	0.323
	Negation	0	0.026
		1	0.052
		2	0.051
		3	0.057
		4	0.050
		5	0.052
	Assertion & Negation	0–4	0.219
